# Importance of Soft Skills in Health Sciences Students and Their Repercussion after the COVID-19 Epidemic: Scoping Review

**DOI:** 10.3390/ijerph20064901

**Published:** 2023-03-10

**Authors:** David Sancho-Cantus, Laura Cubero-Plazas, Marta Botella Navas, Elena Castellano-Rioja, Montserrat Cañabate Ros

**Affiliations:** 1 Department of Nursing, Catholic University of Valencia, 46007 Valencia, Spain; 2 Peset Department, Catholic University of Valencia, 46017 Valencia, Spain; 3 Psychiatry Unit, Clinic Hospital of Valencia, 46010 Valencia, Spain

**Keywords:** soft skills, social skills, nursing students, COVID-19

## Abstract

Soft skills (SKs) are skills related to the interaction among people and their way of dealing with tasks. Increasingly valued in the workplace, they are especially relevant in health professionals due to the importance of the relationship among them and their patients and families. Given their importance, the university training of healthcare professionals must promote the development of SKs. The COVID-19 pandemic has been a turning point in many areas, changing the learning process and, even more, the use of these soft skills as a fundamental ingredient in human relationships. The aim of this study was to analyse the available evidence regarding SKs in health science students, specifically nursing students, and to describe whether there is a worsening in the development of such skills after the COVID-19 pandemic. According to the PRISMA-ScR methodology for systematic reviews, this study included articles on social skills and possible changes in these skills as a consequence of the pandemic in health sciences students The results highlight the importance of these emotional competences for future nurses, being particularly relevant for communication and emotional self-awareness and showing their influence on academic aspects, such as academic performance or mental health and coping skills. A major limitation of the present study was not considering aspects such as compassion or empathy. However, the novelty provided by this work is the analysis of the changes in SKs produced as a consequence of the pandemic. It is definitely clear that there is a need to enhance emotional intelligence, and thus soft skills, in future health professionals.

## 1. Introduction

Soft skills (SKs) are defined as the group of skills acquired by a person that facilitate the optimization of their own performance [1]. Currently, there are numerous studies carried out in relation to SKs, which are a priority in research and policy implementation in the European Union (EU) as a whole, [2,3]. The European Union specifies, in relation to its Sustainable Development Goals, the following. By 2030, there will be a substantial increase in the number of young people and adults who have the necessary skills, in particular technical and vocational skills, for decent work and entrepreneurship [4]. Projects, such as “Soft Skills” [5], financed by EU funds, highlight the relevance of these types of soft skills in the current context.

Training in these skills is of increasing interest in higher education in order to adapt students to an increasingly demanding labour market [6,7]; they are also known as 21st century competencies or social–emotional skills [8,9]. These skills are particularly important in health science professionals, due in part to the nature of their profession, which involves interpersonal contact with patients and families [10]. These professionals are required to have a technical background that includes reasoning and critical judgment, as well as competence in areas such as communication, conflict resolution, negotiation, and decision-making, among others [11,12]. SKs are considered a set of skills necessary for professional performance and the relationship with the environment, and they contribute to the acquisition of social competence by knowing and dealing with the social environment in an effective and adaptive way [13,14,15]. There has been a decline in students’ empathy and understanding of the patient’s situation in undergraduate medical education, and an overly technical approach has replaced a more spontaneous and humane attitude [16]. 

Traditional medical education does not formally include soft skills, such as ethics, professionalism, or communication [17], and relatively few studies describe protocols for professionals to act in stressful situations in clinical practice [18]. Better communication between doctor and patient generates trust, favours the therapeutic bond, and thus improves patient adherence to treatment. It has also been reported that it decreases practitioner mistakes, which reduces conflict and litigation, and it ultimately improves healthcare outcomes.

Despite these limitations, it has been shown that effective communication can be easily taught and practiced. In fact, a recent study has described a theoretical model for working with SKs in medical students based on issues such as patient safety. The SECTORS model describes a situated cognition mode of acquiring these competencies through simulated learning [19].

Among the SKs, communication is an essential element that contributes to improving the quality of care provided by healthcare professionals. Interest in this subject is not recent. As early as 1999, the Accreditation Council for Graduate Medical Education (ACGME) approved six general competencies that postgraduate residents must demonstrate. During the same year, the American Board of Medical Specialties (ABMS) also adopted the same competencies for practicing physicians [20].

Consequently, it also determines patient satisfaction, which directly influences patient recovery [21]. For this reason, communication skills play an important role in the training of health science professionals [22]. Socioemotional competences include aspects, such as awareness of feelings, respect for others, or seeking shared solutions to problems [23].

In order to establish an adequate therapeutic relationship with patients, and at the same time apply effective care strategies, the emotional management of healthcare professionals is crucial; this is related to those aspects related to their ability to understand their own and others’ emotions, to express them correctly, and to know how to put themselves in the patient’s place [15]. Despite the growing interest in the development of SKs in health sciences higher education, there is still little knowledge about how students can effectively acquire these skills in the context of their academic training [6]. Working on these skills will be an essential element for further improving the quality of care provided to patients, as well as patient satisfaction with the attention received from healthcare professionals. Within the SKs, empathy, flexibility, active listening skills, communication, and negotiation strategies can be developed or strengthened through training programmes [24,25]. The outbreak of the COVID-19 pandemic has led to evident and confirmed alterations in the social context, directly affecting social skills in general, and SKs in particular. The reduced contact with other people has led to a deterioration in communication skills involved in human relations [26,27]. The clinical practices of medical and nursing students were drastically affected by the pandemic, resulting in training deficits that affected the acquisition of competencies [28]. 

Social distancing measures imposed by the World Health Organization (WHO) abruptly changed social interactions, leading to different behavioural responses. This fact has undoubtedly marked a turning point and has accelerated the digitalisation of advanced society and the implementation of learning mechanisms that are complementary to those currently in use. For all the reasons previously mentioned, there is a need to develop soft skills to respond effectively to the various situations that may arise in a clinical setting [3,14], including other competences, such as self-control, stress management, or responsibility. 

The aim of this study was to analyse the available evidence on SKs in health science students, specifically in nursing students. Additionally, it aimed to describe whether there is a worsening in the development of these skills after the COVID-19 pandemic.

## 2. Materials and Methods

### 2.1. Study Design and Procedure

A scoping review (SR) was conducted, an appropriate methodology for the objectives proposed in this research, as the aim was to synthesise a specific aspect within an area of evidence, such as health sciences. The methodology used was that proposed by Arksey and O’Malley [29], which consists of the following sequence: (i) identification of the research question; (ii) identification of relevant studies; (iii) selection of studies; (iv) data collection; and (v) communication of the results obtained. The PRISMA protocol for systematic reviews (PRISMA-ScR) was applied [30].

The databases search was conducted between September and October 2022 within the following ones: APA PsycINFO, ERIC, Psychology, and Behavioural Sciences Collection and in PubMed; and a search strategy was developed based on the protocols established by the Joanna Briggs Institute [31,32]. The search terms were health sciences OR nursing students, emotional competences, social skills OR soft skills, interaction or social behavior or social competence, communication skills. Table 1 shows the search equations used and the results obtained.

The study of the articles included in the SR was conducted by means of a content analysis of the themes developed in the selected papers, through a critical reading of the different texts. To carry this out, the articles were coded and grouped into categories, including soft skills and non-technical competencies or interventions during the COVID-19 pandemic.

Filters used to refine the search were: texts published from 2020 to 2022, in either English/Spanish/Portuguese language and which were systematic reviews, randomised clinical trials, cross-sectional, or longitudinal studies.

### 2.2. Eligibility Criteria

Eligible articles met the following inclusion criteria: peer-reviewed texts on soft skills in nursing and/or health sciences students. Opinion articles, academic papers, or articles, whose subject matter was different from the topic under study, were excluded.

### 2.3. Analysis and Synthesis of the Obtained Information 

The following information was extracted from the studies included in the SR: authors, study design, research question/main objective, sample characteristics, method used, and conclusions.

## 3. Results

### 3.1. Overview of Studies

**Figure 1:**
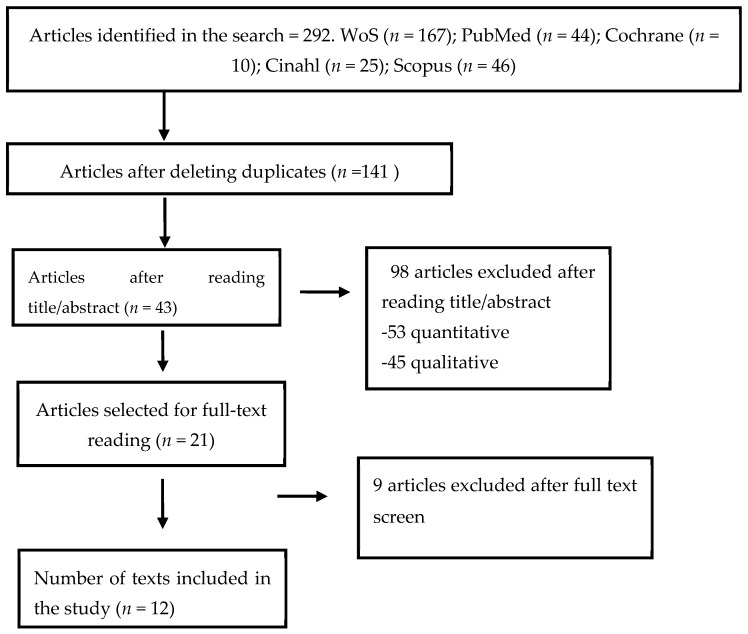
PRISMA flow diagram for study selection.

A total of 292 articles were obtained, with 141 remaining after the elimination of duplicates. Among these, 98 records were excluded after the screening process, since they did not meet the inclusion criteria (Figure 1). After assessing the eligibility of the full text of 21 articles, 12 were included in this scoping review.

### 3.2. Importance of Soft Skills in Nursing

All the texts consulted agree on the importance of emotional competences in the personal and professional development of future nurses [33,34,35,36,37,38,39,40,41,42,43,44] (Table 2). Some authors, such as Kim [33] or Chew [34], evaluated the influence of emotional intelligence (EI) on students’ academic performance and highlighted communication as the main element within EI. Both authors postulated that social–emotional competencies (SEC) acted as predictors of academic performance. According to Kim’s study [33], communication skills were found to affect students’ clinical performance, and critical thinking and problem solving were most strongly correlated with SECs. Chew [34], on the other hand, found an inversely proportional relationship between good socialisation and academic performance in his study. Laari [35], Sarrión-Bravo [36], and Choi [37] suggested, in their studies, that working on soft skills in the classroom improved subsequent contact with the patient, very similarly to the work performed with simulation.

Exploring which emotional competencies emerge as the main ones in nursing students, Waite [38] analysed factors, such as self-awareness, self-management, cognitive factors, social awareness, and social management; and they concluded that the most important ones seemed to be competencies related to emotional self-awareness, emotional self-control, or leadership skills. Nursing students were able to better interact with others to the extent that they recognised and were aware of their own emotions. Ok [39] highlighted leadership or communication skills as key competencies in nursing students. Tanaka [40] studied the impact of social skills in international nursing students and stated that cultural and social factors play an important role in the development of emotional competence in students.

### 3.3. Soft Skills during the COVID-19 Pandemic: What Is Changing?

Kamysbayeva [41] pointed out the deficit in the management of soft skills during the beginning of the pandemic, in which online technologies were effective for working on technical skills (Technical skills complement soft skills and are related to aspects such as clinical reasoning, judgment, and professional expertise. Non-technical skills refer to the management of situations in relation to other people), however not with regard to soft skills. Other authors, such as Soto-Rubio [42], Barros [43], or Utvaer [44], found that EI was shown to be a protective factor against psychosocial risks in nursing professionals, especially work-related stress, and also in relation to mental health in the general population. Mental health was reported to have worsened in the population during the COVID-19 pandemic, especially among women and university students [45]. Stress and anxiety emerged as the main disorders [46,47,48,49], and deficits in soft skills had a negative influence on coping and managing these situations. Nowadays, this deficit is beginning to be perceived both in university students and later in those who obtain an academic degree, and interest in the development of soft skills is growing, as these skills are highly valued by employers [50].

## 4. Discussion

Currently, there are few studies that analyse the importance of SKs for nursing. 

To provide quality health care, not only technical training is required, but also competence in soft skills, such as communication, critical thinking, and group management, among others [51,52]. Most studies agree on this point, although there is no consensus on how these skills should be developed in students [53,54,55]. The American Association of Nursing Councils established new guidelines for nursing education in 2021 in a report entitled The Essentials: Core Competencies for Professional Nursing Education [56]. This report outlines 10 domains and the competencies related to each domain, considered to be the essence of the nursing profession. In Europe, it was the Bologna Declaration in 1999 that laid the foundations for a new training model based on competences, through the Tuning Educational Development initiative [57]. These competences have been related to improved academic performance of students, in line with Kim et al. [33], Chew [34], and Jo [58], supporting the need for their development among nursing students. The practice of SKs in educational centres would facilitate the development of professional competencies necessary in the interaction with patients and relatives, such as communication skills or the improvement of the climate and effectiveness in work teams [2]. An underlying issue that is certainly of great interest in this field is the attitude towards SKs of students in health sciences, which has been investigated by several authors with similar results: students did not have a clear idea about the importance and impact of these skills for their future professional performance [59,60,61]. These findings deserve more attention and further study, and therefore this topic is proposed for future lines of study.

The COVID-19 pandemic has posed a major challenge to the mental health and emotional wellbeing of the population, and the numerous psychological consequences caused by the pandemic can be seen in the increased demand for care. Available evidence indicates that soft skills training provides tools for coping with such disturbances and improving adaptation and resilience [62,63,64,65]. Recent studies have observed the benefits of IE during the pandemic, including reduced intensity of negative emotions and lower prevalence of anxiety and depression [66,67]. No studies are currently available to assess the worsening of SKs development in health science students and professionals as a consequence of the current COVID-19 pandemic, and it is therefore proposed that this line of research should be conducted in the future. The main conclusion of this study is the need to improve SKs among health science students and the potential benefits that such improvement would entail. 

This research presents some limitations. Firstly, the number of articles found is scarce, since, as mentioned previously, there are few recent bibliographical references on this subject in the field of nursing. On the other hand, most of the publications found on the effects produced by the recent pandemic caused by COVID-19 focus mainly on mental health in general, and not that much on specific competences or skills. Finally, the search has mainly focused on SKs, omitting other important competences, such as socio-emotional ones. Therefore, it is proposed to continue this line of research with the aim of further exploring this subject in future studies.

## 5. Conclusions

Among its multiple consequences, the recent SARS-CoV-2 pandemic has led to alterations in the mental health of the population and behavioural changes that include significant deficits in the acquisition and development of SKs.

As a result, SKs currently present a challenge for both the health and educational systems. Efforts are being made by authorities to implement programs to develop and improve these skills among students from the earliest stages of schooling. It is expected that these competencies will become a natural part of the syllabus in the future.

In the health sciences field, the need to work on these types of competences is particularly evident, as their numerous benefits in the clinical context of patient and family care have been highlighted. These SKs improve patients’ and their families’ perceived satisfaction with the care provided, the quality of the care itself, and act as a protective factor against mental health disorders, such as work-related stress or burnout. Competencies such as conflict resolution, teamwork, and effective communication are not only necessary for personal development, but also for professional training. Continuing education is crucial for students and professionals in the health profession to develop a positive attitude towards SKs and progressively improve their clinical competence.

## Figures and Tables

**Table 1:** Search strategies in the different databases.

Search Strategies	PubMed	Cochrane	WoS	Cinahl	Scopus
(soft skills or people skills or social skills or communication skills) AND health science students	44			8	
(social skills or social interaction or social behaviour or social competence) AND health science students				17	
social skills in All Text AND health science students in Title Abstract Keyword		10			
soft skills (All fields) AND nursing (All fields)			167		
(TITLE-ABS-KEY (soft AND skills) AND TITLE-ABS-KEY (nursing))					46

**Table 2:** Importance of soft skills in nursing after the COVID-19 pandemic.

Author/Sample	Study Design	Aim	Outcome Measurements	Conclusions	Country/Study Setting	Tool
Kim et al. [33] 195 (176 female)	Cross sectional	To investigate the correlations between SEC and AA among nursing students	General characteristics, levels of critical thinking disposition, self-directed learning, creativity, emotional intelligence, problem-solving, collaboration, and academic achievements were collected via self-reported questionnaire	Elements of SEC *, including critical thinking disposition, problem-solving, collaboration, self-directed learning, creativity, and emotional intelligence should be fostered to enhance the academic performance of nursing students, in that order	South Korea. nursing students on clinical placement	Yoon’s Critical Thinking Disposition (YCTD) instrument for nursing students; Wong and Law Emotional Intelligence Scale (WLEIS); clinical competency measurement tool for students
Chew et al. [34] 163 final-year medical students	Cross-sectional study	To examine the effect of EI and social management on academic performance	Negative relationships might exist between emotional–social intelligence and academic success in undergraduate medical students	A different collection of social skills and SM EI could be constructive towards academic achievement in medical schools	Medical students in a public medical school in Malaysia	Mayer-Salovey-Caruso Emotional Intelligence Test (MSCEIT)
Laari et al. [35] 109 students	Quantitative research	To explore nursing students’ understanding of the concept of soft skills and to acquire their perception on the need for soft skills training to promote quality nursing care	They furthermore agreed that soft skills should be part of the training that student nurses receive during their professional training	There is a need for nursing students to be educated in soft skills, and this will enhance their job performances in the clinical environment and improve the way in which they communicate with their clients	Nursing training college in the upper east region of Ghana	No se especifica el nombre del cuestionario
Sarrión-Bravo et al. [36] 21 studies	Qualitative study	To define a catalogue of learning outcomes for spiritual and emotional care for undergraduate nurses	An amount of 65 learning outcomes: 14 for assessment and diagnosis; five for planning; 17 for intervention; four for evaluation and quality; eight for communication and interpersonal relationship; and 17 for knowledge and intrapersonal development	The academic curriculum can include these learning outcomes to help undergraduate nurses in the process of acquiring knowledge, skills, and attitudes in spiritual and emotional care	Degree in nursing in Spain students	Delphi Method
Choi et al. [37] The experimental group included 45 students, and the control group included 42 students	Quasi experimental study	To verify the communication skills training for nursing students by using a video clip on a smart phone	The experimental group improved more significantly than the control group in communication competence and emotional intelligence.	Using a video clip on a smart phone is helpful for communication teaching method	Nursing students in two universities in South Korea	Cuestionario sobre comunicación (No se especifica el nombre del cuestionario)
Waite et al. [38] 14 nursing students (female)	Descriptive	Assessing emotional and social competencies in a sample of nursing students	The Emotional and Social Competency Inventory, University Edition (ESCI-U), was used to assess participants’ skills in recognizing, managing, and motivating their own emotions, as well as their social ability to be cognizant and respectful of others’ feelings	Statistical significance was found with three core areas: emotional self-awareness, emotional self-control, and inspirational leadership	Philadelphia, Pennsylvania pre-licensure nursing students	Emotional and Social Competency Inventory
Ok et al. [39] 190 nursing students	Descriptive research	The factors influencing clinical competence were self-leadership (ß = 0.33) and communication skills (ß = 0.22), and the explanatory power of these factors was 31.8%	It is important to improve the clinical competence by enhancing self-leadership and communication skills of nursing students		Three universities located in two cities in the Republic of Korea	Global Interpersonal Communication Competency Scale (ICC); Wong and Law Emotional Intelligence Scale (K-WLELS)
Tanaka et al. [40] 140 questionnaires	Descriptive	Assessing the importance of social skills in a group of international nursing students	The skills were divided into the following four categories: (a) skills used in Japan and recognized as being used in home countries; (b) skills used in Japan but not recognized in their own countries; (c) skills recognized in their home countries but not used in Japan; and (d) neglected skills	The degree of use of each skill varied	International students in Japan	Cross-cultural social skills for international students in Japan
Kamysbayeva [41] 300 graduate students	Qualitative research	To study modern problems and opportunities for the implementation of e-learning in higher educational institutions in the context of the COVID-19 pandemic	Online learning is an efficient tool for the development of hard skills while being less effective for the improvement of soft skills	E-learning format is an effective methodology for the development of technical and digital skills of students	Almaty, Kazakhstan 300 graduate students	Qualitative method
Soto-Rubio et al. [42] 119 nurses	Cross-sectional	To analyse the relationships between EI, relationships and psychological risks in the Spanish context during the pandemic	Protective effect of emotional intelligence against the adverse effects of psychosocial risks	The emotional intelligence of nurses, in particular the emotional attention dimension, can be a risk factor for some psychosocial risks	Spanish nurses	The Trait Meta-Mood Scale; The UNIPSICO Battery; The Frankfurt Emotional Work Scale; The Questionnaire for the Assessment of Workplace Burnout Syndrome
Barros et al. [43] 923 university students	Cross-sectional study	To analyse the mediator role of emotional intelligence and social support on university students’ mental health, taking into consideration the role of gender differences	Being connected with and supported by friends and family members during the COVID-19 pandemic worked as a coping mechanism, this being a protective factor of mental health	Importance of developing continuous psychological interventions among students and evaluating their mental health and wellbeing over time.	Portugal university students	The WLEIS emotional intelligence scale; The DASS-21, Depression, Anxiety, and Stress Scale; The MSPSS, Multidimensional Scale of Perceived Social Support
Karin et al. [44] 305 students	Cross-sectional study	To investigate the associations between peer support, teacher support, emotional state, and perceived competence in nursing students during the pandemic	Teacher support had a significant direct effect on perceived competence, while peer support almost had a significant direct effect	During the COVID-19 pandemic, both peer and teacher support can significantly support students’ competence development	An amount of 29 nursing students at a large university in Norway	Perceived Competence for Learning scale

* SEC = socio-emotional competence (s); AA = academic achievement; EI = emotional intelligence; SM = social management.
